# Molecular mechanism of oil induced growth inhibition in diatoms using *Thalassiosira pseudonana* as the model species

**DOI:** 10.1038/s41598-021-98744-9

**Published:** 2021-10-06

**Authors:** Manoj Kamalanathan, Savannah Mapes, Jessica Hillhouse, Noah Claflin, Joshua Leleux, David Hala, Antonietta Quigg

**Affiliations:** 1 Department of Marine Biology, Texas A&M University at Galveston, Galveston, TX 77553 USA; 2 Department of Oceanography, Texas A&M University, College Station, TX 77845 USA; 3 Present Address: Virginia Institute of Marine Science, Gloucester Point, VA 23062 USA

**Keywords:** Biochemistry, Microbiology, Molecular biology, Physiology, Plant sciences, Limnology

## Abstract

The 2010 Deepwater Horizon oil-spill exposed the microbes of Gulf of Mexico to unprecedented amount of oil. Conclusive evidence of the underlying molecular mechanism(s) on the negative effects of oil exposure on certain phytoplankton species such as *Thalassiosira pseudonana* is still lacking, curtailing our understanding of how oil spills alter community composition. We performed experiments on model diatom *T. pseudonana* to understand the mechanisms underpinning observed reduced growth and photosynthesis rates during oil exposure. Results show severe impairment to processes *upstream* of photosynthesis, such as light absorption, with proteins associated with the light harvesting complex damaged while the pigments were unaffected. Proteins associated with photosynthetic electron transport were also damaged, severely affecting photosynthetic apparatus and depriving cells of energy and carbon for growth. Negative growth effects were alleviated when an organic carbon source was provided. Further investigation through proteomics combined with pathway enrichment analysis confirmed the above findings, while highlighting other negatively affected processes such as those associated with ferroxidase complex, high-affinity iron-permease complex, and multiple transmembrane transport. We also show that oxidative stress is not the primary route of negative effects, rather secondary. Overall, this study provides a mechanistic understanding of the cellular damage that occurs during oil exposure to *T. pseudonana*.

## Introduction

Phytoplankton responsible for 43.5 Pg C yr^−1^ of the primary productivity on this planet^1^, of which, nearly 40% is accounted for by diatoms^2^. In aquatic environments, they are often exposed to stressors including nutrient limitation, heavy metals, oil, UV, elevated CO_2_ levels and ocean acidification. Exposure to these stressors might act as selection forces against certain species, while benefitting others, thereby changing the phytoplankton community composition^3–5^. Such selection events can therefore have cascading effects on other factors such as nutrient cycling, phytoplankton-bacteria interaction(s), primary and secondary productivity and bacterial community composition. Toxicological studies conducted so far include investigating effects of metals such as copper and cadmium^6–9^, pH^10,11^, CO_2_^12–14^, UV^15,16^, light stress, nutrient stress from nitrogen, phosphorus, or silica limitation^17–21^, and components of oil^10,22–27^. In recent years, emergent pollutants including engineered nanomaterials^28^, persistent organic pollutants^29^ and more recently, nanoplastics^30^, have been also found to be toxic to many phytoplankton. Collectively these factors underscore the importance of examining the effects of stressors on phytoplankton growth and primary production. The 2010 Deepwater Horizon oil-spill exposed phytoplankton of Gulf of Mexico to nearly 4.1 million barrels of oil. The effects of this oil exposure changed the phytoplankton community^31^, affecting their composition, primary productivity and overall growth^32^. The exact mechanism behind how oil exposure affects growth and photosynthesis and therefore altering the phytoplankton composition remains unknown.

Several studies focusing on the effects of oil has been conducted since the Deepwater Horizon oil spill^33^ and references therein, but many questions remain. Often considered as a representative for marine diatoms, *Thalassiosira pseudonana* (CCMPS 1335) is one of the most commonly studied species, with its genome sequenced^34,35^. Transcriptomic and metabolic studies on *T. pseudonana* have suggested that exposure to certain components of oil, such as Benzo (a) pyrene (BaP), or a mixture of polycyclic aromatic hydrocarbons (PAH), in addition to oxidative stress, leads to inhibition of silicon uptake and fatty acid synthesis^22–25^. Similarly, oxidative stress caused by oil exposure was also noted by Ozhan et al.^36^. The fate of fatty acid synthesis however remains unclear in *T. pseudonana* upon oil exposure*,* with Carvalho et al.^24^ suggesting an increase in lipid synthesis, while Carvalho et al.^25^ showed downregulation of lipid synthesis associated genes. Batch experiments revealed that the exposure to oil results in two different growth phases in exponentially growing *T. pseudonana*^27^. During the first phase, growth is inhibited in the presence of oil, followed by a second phase where growth recovers as the oil concentration declines. Polysaccharide synthesis was shown to play an important role in the recovery of growth of *T. pseudonana* once the oil concentration nears zero^27^. The mechanistic action of oil on the growth of *T. pseudonana* during the first phase remains unknown. Previous study by Babu et al.^37^ proposed a model in which 1,2‐dihydroxyanthraquinone (1,2‐dhATQ) inhibits electron transport at the site of Cytochrome b6/f complex in duckweed *Lemna gibba* exposed to oil. Srivastava et al.^38^ suggested that benzoquinone (which is often the photoproduct of PAHs) can lower the electron transport between photosystem II and I by directly accepting electrons from PSII. Kottuparambil et al.^39^ showed that increased reactive oxygen species (ROS) accumulation at the photosystem due to exposure to PAH anthracene can lead to reduced photosynthetic electron transport in freshwater flagellate alga *Euglena agilis*.

Studying the response of phytoplankton to environmental stressors is of vital interest as oil spills and other pollutants alter community composition and survivorship, and ultimately higher trophic levels. In this study, we endeavor to establish a clear understanding of how exposure to oil affects the growth and physiology of phytoplankton using the model species *T. pseudonana*. Using a combination of traditional laboratory experiments and modern proteomics approach, we sequentially tested the proposed hypotheses which include (1) oil exposure leads to inhibition of silicon uptake and fatty acid synthesis^22–25^, (2) oil exposure leads to oxidative stress and therefore intracellular damage^22–25,36^, and (3) PAHs, the biological toxic component of oil, affect the electron transport between photosystem II and I by directly accepting electrons from PSII^37,38^. The results presented provide direct and detailed mechanistic evidence of how oil exposure affects growth and photosynthesis in *T. pseudonana* and therefore an explanation of how oil spills could negatively impact diatoms and other sensitive species and ultimately alter the phytoplankton community composition.

## Results

**Figure 1:**
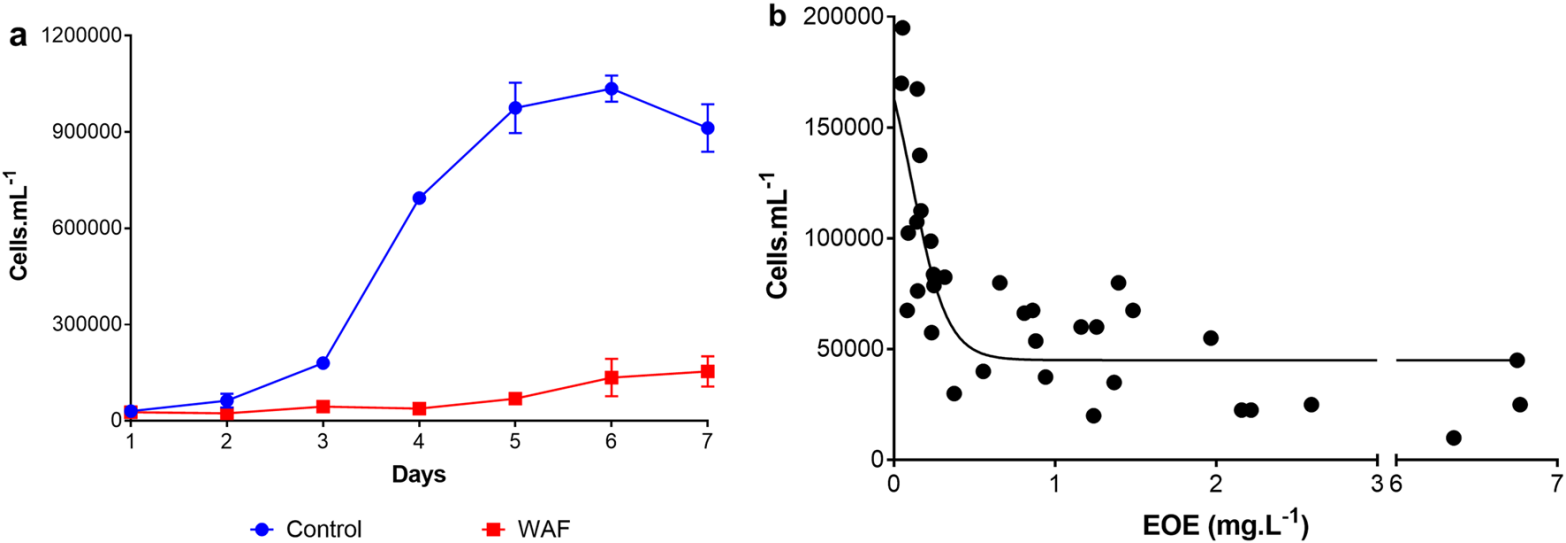
Growth response of *T. pseudonana*. (**a**) average daily cell counts of *T. pseudonana* ( ±) in Control and WAF (n = 3), (**b**) Cell counts of *T. pseudonana* under different estimated oil equivalents (oil concentrations).

Exposure of *T. pseudonana* to a water accommodated fraction (WAF) of oil resulted in significantly lower cell counts throughout the growth cycle compared to the Control (student t-test, *p* = 0.0149) (Fig. 1a). WAF cultures did not increase in cell numbers during the first four days of the experiment (first phase), followed by an increase at the end of the experiment (second phase). Figure 1b showed an inverse pattern of cell abundance versus hydrocarbon concentration measured as estimated oil equivalents (EOE), with cell numbers increasing with decreasing EOE concentration. To gain an understanding of the molecular mechanism inhibiting the growth of *T. pseudonana* in WAF during the first phase, we first examined the cell structural physiology after 48 h to determine if there was morphological damage. Significantly larger cells both by volume and surface area were measured in WAF treatments compared to the Control (student t-test, *p* < 0.0001) (Table 1). Further examination of the morphology showed that *T. pseudonana* had a nearly 1.75 fold increase in height under WAF compared to the Control, and a 1.17 fold increase in radius (student t-test, *p* =  < 0.0004) (Table 1). Analysis of silica content of the cells showed significantly higher silica per cell and surface area of *T. pseudonana* in WAF relative to Control (student t-test, *p* =  < 0.0001) (Table 1).

**Table 1:** Cellular morphology of *T. pseudonana*. Table summarizing morphological parameters such as cell volume, cell surface area, radius, height, silica content per cell and surface area of *T. pseudonana* cells grown under Control and WAF conditions (n = 3).

	Control	WAF
Cell volume (µm^3^)	116.66 (± 2.669)	277.43 (± 17.867)
Surface area (µm^2^)	130.69 (± 2.581)	235.75 (± 10.892)
Radius (µm)	2.69 (± 0.053)	3.15 (± 0.039)
Height (µm)	4.950 (± 0.037)	8.668 (± 0.423)
Silica content.Cell^−1^ (mg Silica. Cell^−1^)	7.00e-05 (± 1.08e-06)	2.70e-04(± 1.95e-06)
Silica content.Surface area^−1^ (mg Silica. µm^2^)	5.36e-07 (± 1.70e-08)	1.15e-06 (± 4.65e-08)

**Figure 2:**
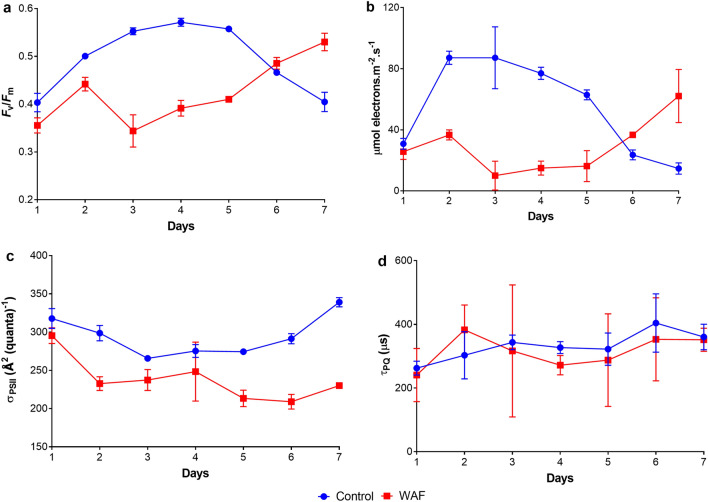
Photo-physiological response of *T. pseudonana*. (**a**) average maximum quantum yield of PS II (*F*_v_/*F*_m_), (**b**) average functional absorption cross-section area of PS II (σ_PSII_; Å2 quanta^–1^), (**c**) average maximum electron transport rates (*r*ETR_max_; µmol electrons m^−2^.s^−1^), and (**d**) average Q_A_ turnover rates of plastoquinone (τ_PQ_; µs) of *T. pseudonana* ( ±) under Control and WAF treatments (n = 3).

An examination of the photo-physiology of *T. pseudonana* showed significantly lower photosynthetic efficiency (*F*_v_/*F*_m_) during the first five days in WAF treatments compared to the Controls (student t-test, *p* = 0.0069) (Fig. 2a). Similar trends were seen for maximum relative electron transport rates (*r*ETR_max_; µmol electrons m^−2^.s^−1^) values (student t-test, *p* = 0.0031) (Fig. 2b). WAF treatments also had significantly lower functional absorption cross section areas (σ_PSII_, Å^2^ quanta^–1^; student t-test, *p* = 0.0023) compared to the cells in the Control treatment (Fig. 2c), however no difference was observed in the rates of plastoquinone (Q_A_) turnover (τ_PQ_, µs; student t-test, *p* = 0.5196) (Fig. 2d). Light harvesting efficiency (α; µmol electrons/µmol photons) also showed trends similar to *F*_v_/*F*_m_ and *r*ETR_max_, with significantly lower values seen in WAF treatment in the first five days (student t-test, *p* = 0.0193) (Fig. S1).

**Figure 3:**
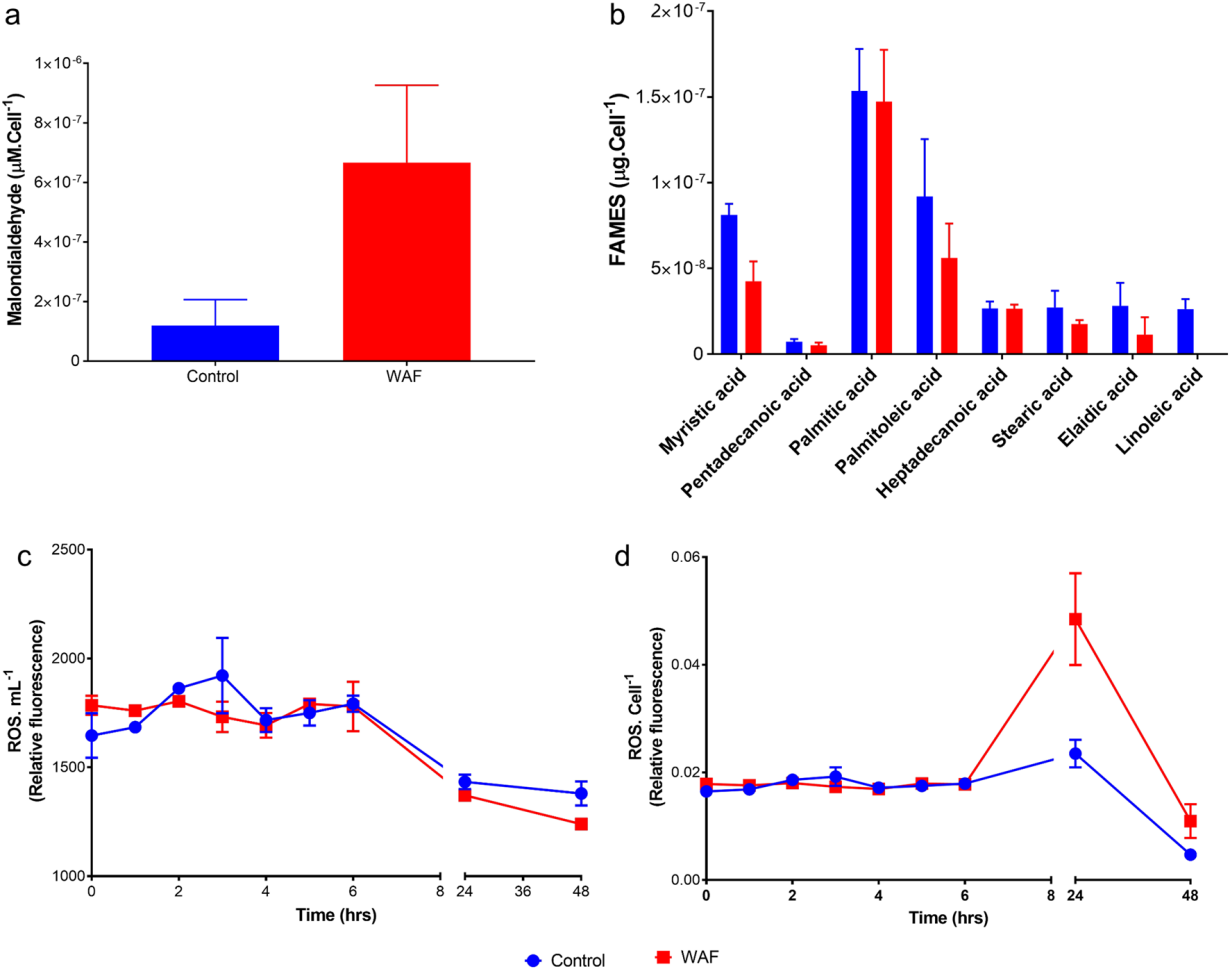
Biochemical response of *T. pseudonana*. (**a**) average malondialdehyde per cell (MDA) after 48 h of incubation, (**b**) average FAME composition per cell of *T. pseudonana* after 48 h of incubation, (**c**) average total reactive oxygen species per mL (ROS.mL^−1^) through time during the 48 h of incubation, and (**d**) average total reactive oxygen species to cell concentration ratio (ROS.cell^−1^) through time during the 48 h of incubation in *T. pseudonana* (± standard deviation) under Control and WAF treatments (n = 3).

To understand the molecular mechanism behind the differences in photo-physiology, we determined ROS and malondialdehyde (MDA) levels in both the treatments after 48 h of exposure. MDA levels were also significantly higher in WAF compared to the Control (student t-test, *p* = 0.0258) (Fig. 3a). An analysis of total ROS levels over a period of 48 h revealed no significant differences between the Control and WAF treatments (student t-test, *p* = 0.4177) (Fig. 3c). Expressing the ROS levels per cell also showed similar pattern for the first six h (student t-test, *p* = 0.9531), however, the values were significantly higher in the WAF treatment compared to the Control at 24 and 48 h time point (student t-test, *p* = 0.0482) (Fig. 3d).

To confirm higher lipid peroxidation (as indicated by MDA levels) in WAF treated cells relative to the Control, we compared the fatty acid methyl ester (FAME) composition in both treatments. We observed lower levels of *total* FAMEs in WAF compared to the Control, however the values were not statistically significant (student t-test, *p* = 0.0833) (Fig. S2). Analysis of FAME components between the two treatments showed variation in fatty acid composition, with no linoleic acid, and significantly lower myristic acid in WAF treatment compared to Control (student t-test, *p* = 0.0073) (Fig. 3b). Lower levels of palmitoleic acid, and elaidic acid were also observed in the WAF treatment compared to Control, however these differences were not significant (student t-test, *p* > 0.1588) (Fig. 3b). Similar levels of pentadecanoic, palmitic and heptadecanoic acid was observed in WAF treatment compared to the Control treatment (student t-test, *p* > 0.1789) (Fig. 3b).

**Figure 4:**
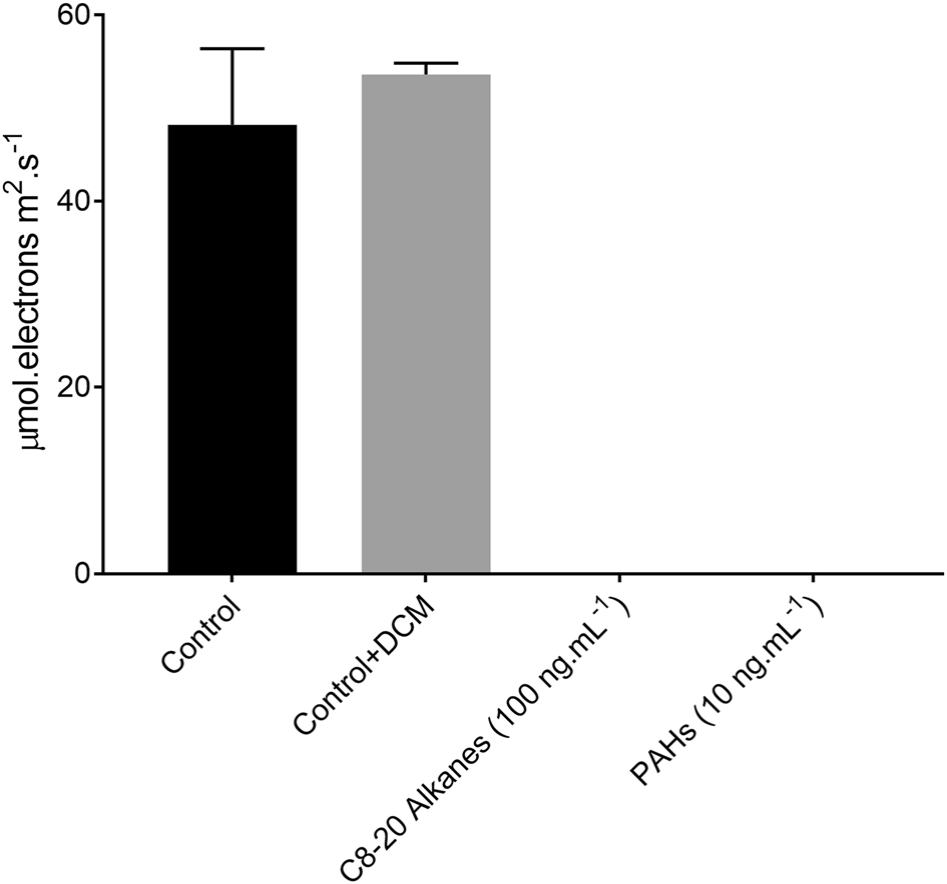
Effects of short-term exposure of alkane, PAH mix, and the solvent DCM on relative electron transport rates of *T. pseudonana* (± standard deviation) after 48 h of incubation under Control and WAF treatments (n = 3).

To determine the chemical nature of oil components affecting the growth and physiology of *T. pseudonana* in the WAF treatment, we measured the photosynthetic electron transport in *T. pseudonana* after exposure to alkanes and PAHs for only 60 min. *r*ETR_max_ were completely inhibited in the presence of both alkanes and PAHs, with no observed photosynthetic electron transport in these treatments (One-way ANOVA, *p* < 0.0001) (Fig. 4). Analysis of nonphotochemical quenching (NPQ) in *T. pseudonana* in both the Control and WAF treatments after 48 h showed no significant differences (student t-test, *p* = 0.2735) (Fig. S3), however, respiration rates were significantly higher in WAF compared to the Control (student t-test, *p* = 0.0398) (Fig. S4).

**Figure 5:**
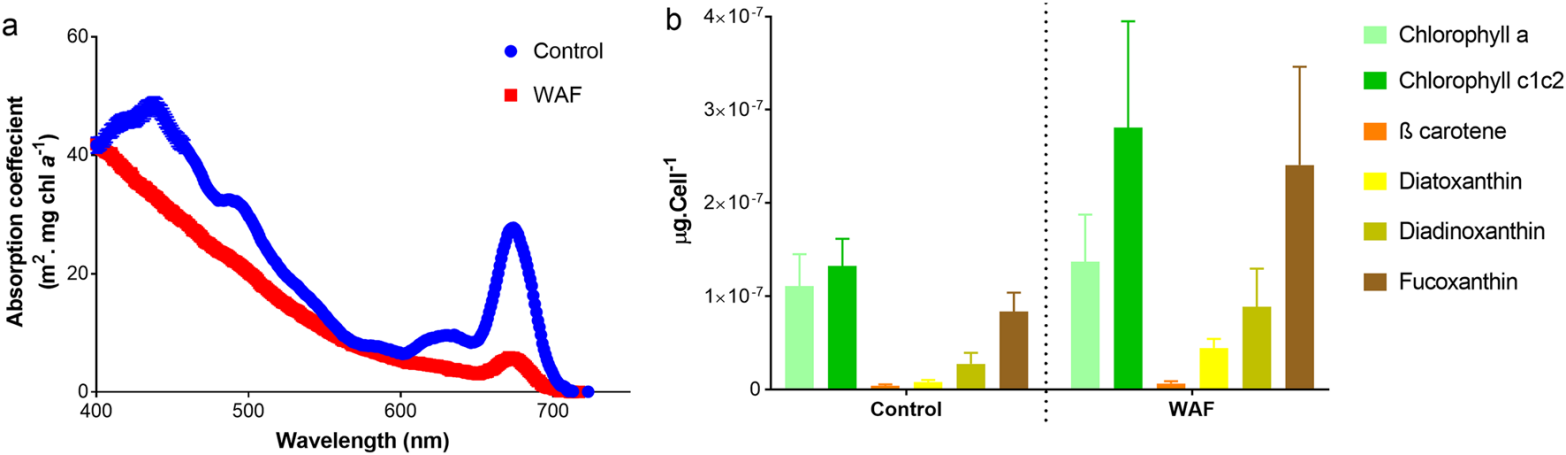
Light harvesting components of *T. pseudonana*. (**a**) average absorption coefficient, (**b**) average pigment composition of *T. pseudonana* (± standard deviation) after 48 h of incubation under Control and WAF treatments (n = 3).

To understand the primary site of action of oil components in *T. pseudonana* leading to inhibition of photosynthesis, we compared both the physiological processes and pigments associated with light harvesting process of photosynthesis. The absorption coefficient, a function of pigment-protein complex associated with the light absorption process of photosynthesis, was significantly lower in WAF compared to the Control (student t-test, *p* < 0.0001) (Fig. 5a). Further analysis of pigment composition in both treatments suggested no significant differences for most pigments (student t-test, *p* > 0.0645) (Fig. 5b), except diatoxanthin, which was higher in the WAF treated cells (student t-test, *p* = 0.0030). In addition, determination of de-epoxidation of the xanthophyll cycle, which controls the dissipation of excess absorbed energy as NPQ, showed no significant difference between the treatments (student t-test, *p* = 0.0967) (Fig. S5).

**Figure 6:**
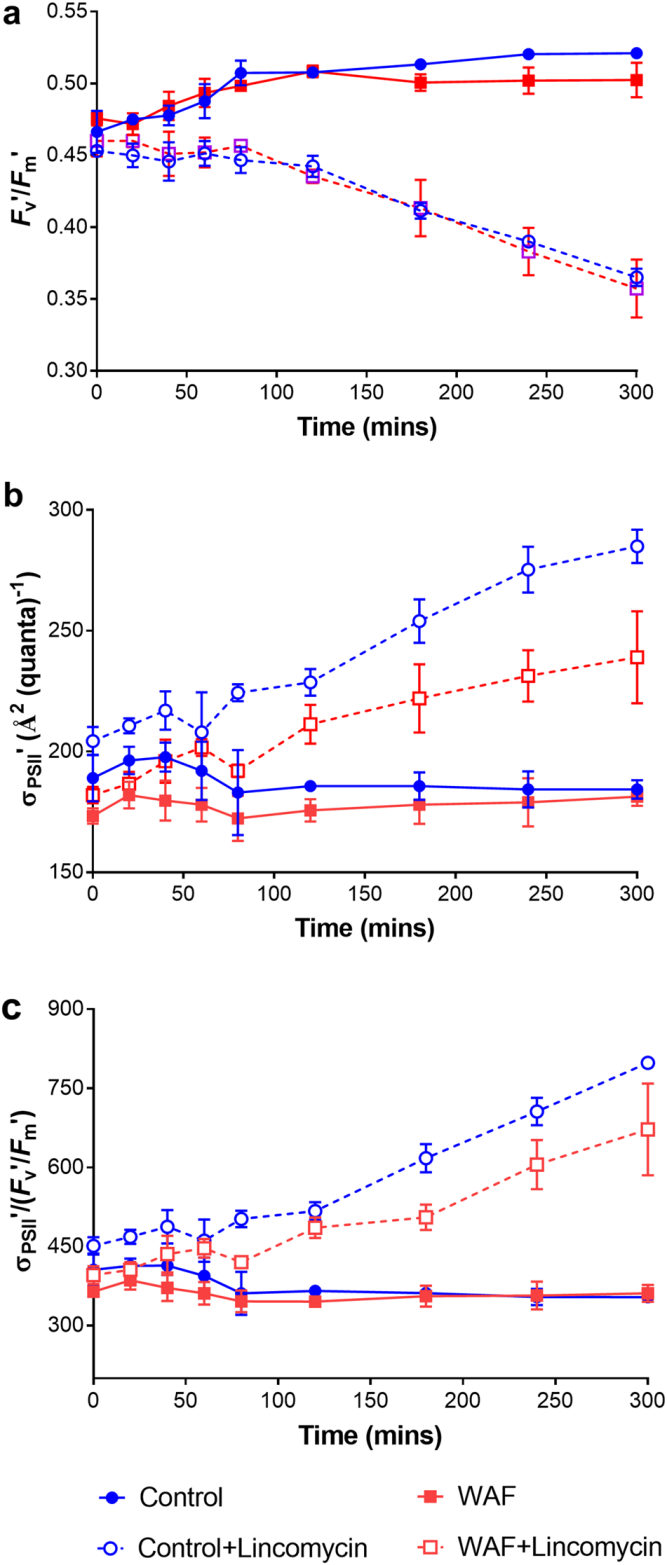
Effect of WAF exposure on the protein components of light harvesting complex. (**a**) average functional cross-section area (σ), (**b**) average quantum yield of PS II (*F*_v_’/*F*_m_’), and (**c**) σ/(*F*_v_’/*F*_m_’) measured in of *T. pseudonana* cultures after 100 min of in Control and WAF conditions, followed by addition of 2 mM Lincomycin.

To test the effects of oil exposure on proteins associated with light harvesting complex, we measured the photosynthetic yield (*F*_v_’/*F*_m_’), the relative functional absorption cross-section area (σ’), and estimated light absorption (σ’/(*F*_v_’/*F*_m_’)) in WAF and Control in the presence and absence of the protein synthesis inhibitor, lincomycin. No significant differences in *F*_v_’/*F*_m_’ values were observed between the Control and WAF or Control + lincomycin and WAF + lincomycin treatment (One-way ANOVA, *p* > 0.9248) (Fig. 6a). However, the *F*_v_’/*F*_m_’ were significantly lower in both Control + lincomycin and WAF + lincomycin compared to Control and WAF treatment (One-way ANOVA, *p* < 0.0138) (Fig. 6a). Significantly lower σ’ was observed in WAF compared to the Control for the first 40 min of measurements (Student t-test, *p* = 0.0045), this difference then began to diminish over time, and the σ’ values were similar by 180 min (Fig. 6b). Presence of lincomycin led to a significant increase in σ’ under both Control and WAF treatments, especially after 60 min (One-way ANOVA, *p* < 0.0050) (Fig. 6b). However, σ’ values were significantly lower in WAF + lincomycin compared to Control + lincomycin (Student t-test, *p* = 0.0002) (Fig. 6b). σ’/(*F*_v_’/*F*_m_’), which is an indicator of light absorption showed trends similar to σ’, with significantly higher values in the presence of lincomycin for both Control and WAF treatment, especially after 60 min (One-way ANOVA, *p* < 0.0074) (Fig. 6c). Lower σ’/(*F*_v_’/*F*_m_’) values were observed for WAF treatment compared to the Control for the first 120 min of measurements (Student t-test, *p* = 0.0261), followed by more similar values afterwards (Fig. 6c). In the presence of lincomycin, WAF treatment had significantly lower σ’/(*F*_v_’/*F*_m_’) values compared to the Control (Student t-test, *p* = 0.0005) (Fig. 6c).

**Figure 7:**
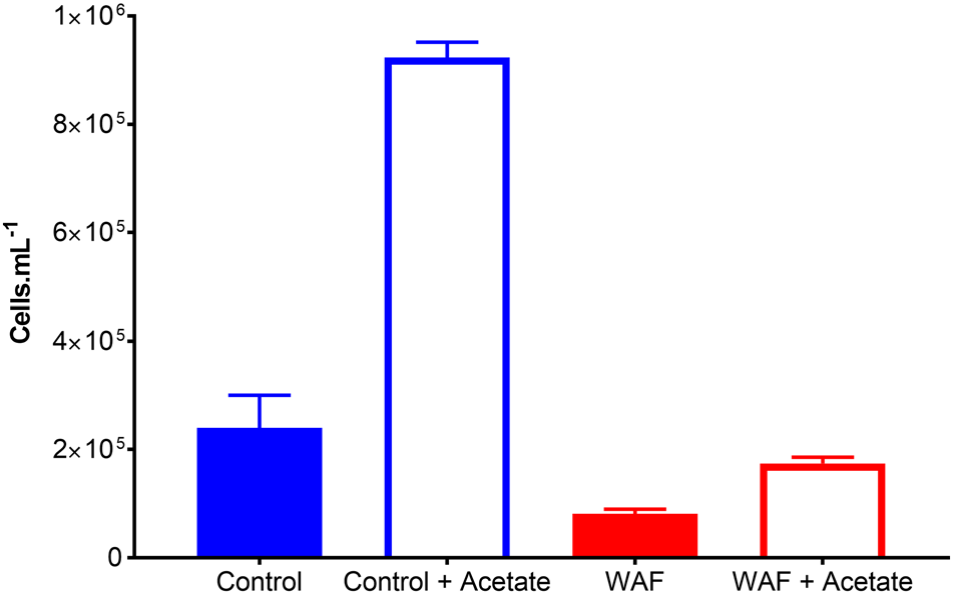
Effect of external organic carbon source (0.5 g.L^−1^) on the growth of *T. pseudonana* (± standard deviation) after 48 h of incubation under Control and WAF treatments (n = 3).

To test the deficiency of carbon and energy supply caused by negative effects of oil exposure on photosynthesis, growth of *T. pseudonana* was compared in Control and WAF in the presence and absence of an external organic carbon and energy source- acetate. The cell numbers were significantly higher in WAF + Acetate treatment compared to WAF only (One-way ANOVA, *p* = 0.02) (Fig. 7). Moreover, the cell numbers in WAF + Acetate were similar to that in Control only treatment (One-way ANOVA, *p = *0.137), although the numbers were significantly lower than Control + Acetate (One-way ANOVA, *p* < 0.0001) (Fig. 7).

**Figure 8:**
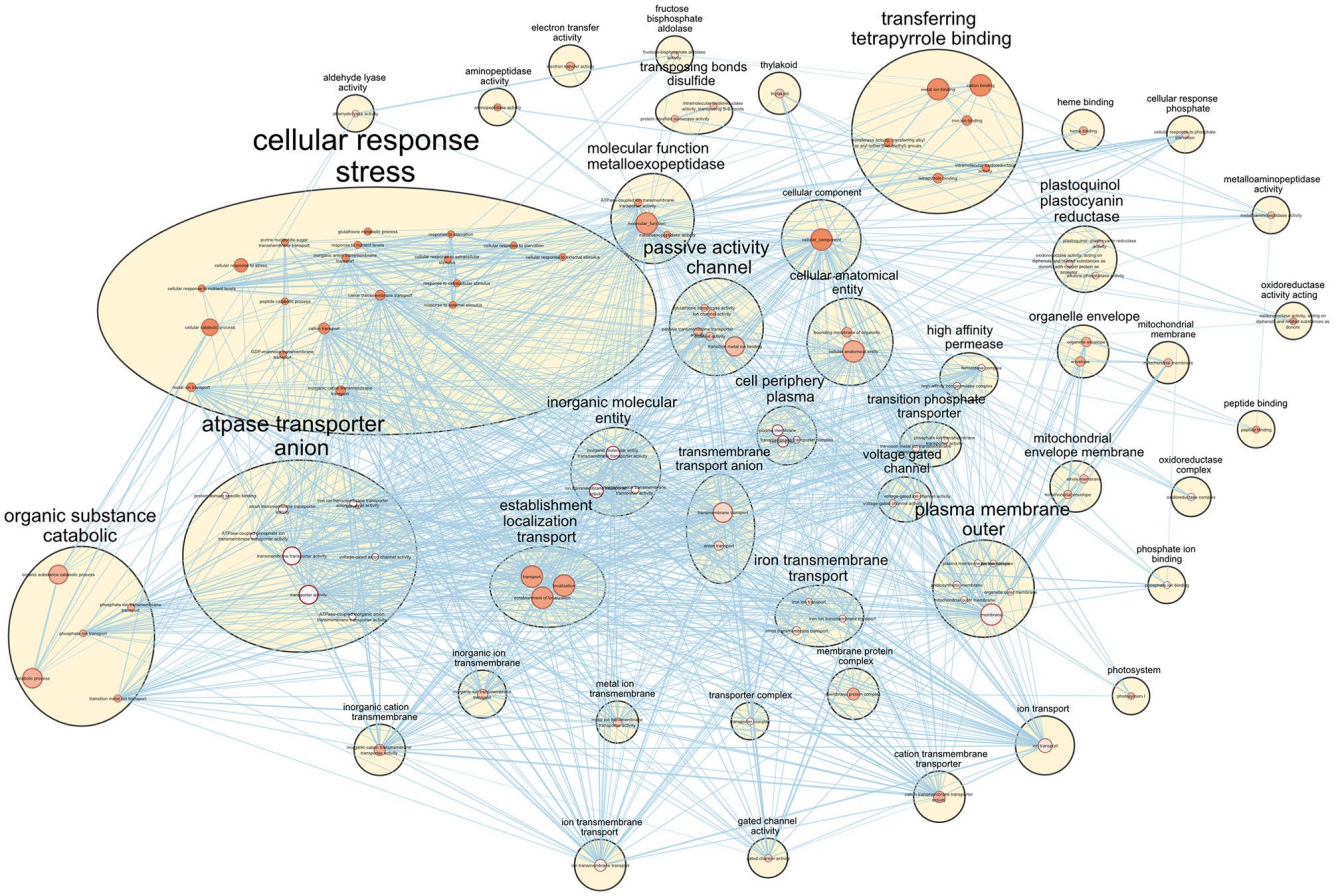
Negatively affected biological functions or gene ontology terms in *T.pseudonana* after 48 h of exposure to WAF relative to Control. The size of the node corresponds to the size of the gene sets and the color of the node ranging from orange-white-blue indicates increasing FDR q values.

Analysis of proteome data revealed 44 significant peptides that were differentially present between Control and WAF treatment (FDR adjusted *p* < 0.05) (Supplementary Table 1). All peptides were present in lower abundance with the exception of THAPSDRAFT_40669 (Ubiquitin-like domain-containing protein, associated with protein degradation), which was threefold higher in WAF treatment compared to the Control (FDR adjusted *p* = 0.04). Pathway enrichment analysis using the 43 peptides found in lower abundance in WAF revealed several biological functions that were downregulated (Fig. 8, Supplementary Table 1). Biological functions associated with photosynthesis, ferroxidase complex, high-affinity iron permease complex, transmembrane transport activity such as inorganic anion transmembrane transporter activity, ion transmembrane transporter activity, transmembrane transporter complex, cell periphery and plasma membrane were top ten significantly affected processes (Fig. S6, Supplementary Table 1). The most significant gene set (KEGG:00,195) (FDR q-value = 4.53241E-06) associated with photosynthesis unfortunately could not be mapped while building the enrichment map. This gene set which was significantly lower in the WAF treatment contained proteins such as THAPSDRAFT_35934 (Cytochrome c domain-containing protein), THAPSDRAFT_270229 (similar to cytochrome b6/f), THAPSDRAFT_BD1611 (predicted to be associated with PS I), THAPSDRAFT_20603 (similar to Oxygen-evolving enhancer protein 3). Performing enrichment analysis for cellular component type revealed organelles such as plasma membrane outer, mitochondrial membrane, thylakoids and cell periphery plasma to be severely affected in WAF (Fig. S6).

## Discussion

Understanding how oil negatively impacts phytoplankton will help explain how oil spills alter their community. While there are several reports on the negative effects of oil on phytoplankton, we still lack a clear understanding on the molecular effects underlying the observations. Here we address a series of sequential hypothesizes to study how oil inhibits the growth and photosynthesis of a model diatom species, *T. pseudonana*. Unlike earlier studies, we measure alterations in growth, photosynthesis, physiology, pigments, proteins, ROS and proteomics in a comprehensive effort to target the mechanistic basis underlying the cellular response.

Growth of this diatom was negatively affected by the presence of oil in a concentration dependent response as observed in previous studies^26,27^, with recovery of growth resuming as soon as oil levels fell to below detection limits (< 0.08 mg/L). Inhibition of silica uptake of *T. pseudonana* was reported by Carvalho et al.^23^; with follow up studies examining the transcriptomics and proteomics^24,25^. We observed a higher silica content per cell and higher cellular surface area (Table 1), in contrast to these studies. This difference can be explained by the measured inhibition of cell multiplication and increased cellular volume of *T. pseudonana* when exposed to oil in the present study. Uptake of silica is a carrier-mediated saturable system that requires energy and is tightly coupled with the cell cycle^41^. Therefore, the decreased expression and abundance of genes and proteins associated with silica uptake as observed in previous studies^22–25^ could be simply due to the growth inhibition caused by oil exposure and the cellular need to conserve energy and cellular resources under such stressful conditions.

To develop a deeper understanding of the mechanism of growth inhibition caused by oil exposure, we analyzed the photo-physiology of the cells when exposed to WAF. We measured lower photosynthetic electron transport rates between PSII and PSI (as *r*ETR_max_). This could be explained by the decreased functional light absorption cross-section area in WAF treated cells relative to the Control, which suggests that the *upstream* process of the light reactions of photosynthesis-light absorption were negatively affected in *T. pseudonana* when exposed to oil. Moreover, no significant change in plastoquinone (Q_A_) turnover rates in WAF treated cells compared to the Control suggests that the *downstream* process of light reactions-linear photosynthetic electron transport from Q_A_ to Q_B_ was largely unaffected. Together these results indicate that the observed lower electron transport rates in WAF treated cells compared to the Control was primarily due to lower light absorbed by *T. pseudonana* when exposed to WAF. To confirm this, non-photochemical quenching and rates of respiration of *T. pseudonana* were measured. Non-photochemical quenching allows the cells to dissipate excess of the absorbed light energy in the form of heat, when the balance of light absorption and light utilization is disrupted in a way that the former exceed the latter^42,43^. Such a scenario occurs if the cells are exposed to higher than optimum light levels, or if the cells are continuing to absorb light when the *downstream* processes such as photosynthetic electron transport or carbon fixation are negatively affected resulting in photo-oxidative damage of the photosynthetic apparatus^44^. In addition, photosynthetic electron transport itself can produce ROS as a byproduct^45^. No significant differences in NPQ values between the Control and WAF treatments suggest that the light absorption was either below or in balance with the *downstream* light absorption led excitation energy utilization processes such as photosynthetic electron transport and carbon fixation.

Babu et al.^37^ suggested that anthracene photoproducts can block photosynthetic electron transport processes by inhibiting the cytochrome b6/f. Previous studies^46,47^ have suggested that photosynthetic electron transport and mitochondrial electron transport processes have components that are structurally homologous, especially cytochrome b6/f. Therefore, if oil components were to inhibit the photosynthetic electron transport, one can expect the same fate for mitochondrial electron transport. However, we observed significantly higher rates of respiration in *T. pseudonana* when exposed to WAF compared to the Control, suggesting the mitochondrial electron transport were unaffected by the presence of oil. However, proteome analysis showed significantly lower abundance of THAPSDRAFT_270229, a protein similar to cytochrome b6/f in WAF, which aligns well with the observation made by Babu et al.^37^. This suggests that photosynthetic cytochrome b6/f were more prone to oil associated damage than mitochondrial. In addition, peptides such as THAPSDRAFT_BD1611 (predicted to be associated with PS I), THAPSDRAFT_20603 (similar to Oxygen-evolving enhancer protein 3) and THAPSDRAFT_35934 (Cytochrome c domain-containing protein) were also found in significantly lower abundance in WAF treatment, suggesting oil associated damage beyond cytochrome b6/f. These findings along with unaffected Q_A_ turnover rates provides further evidence that the negative effects of oil on photosynthesis may not be due to the PAH components of oil accepting electrons from the reduced photosystems as previously hypothesized^38,39^, rather by a direct damage of electron transport proteins by oil interaction^37^. The lower functional absorption cross-section area and similar NPQ observed in WAF compared to the Control indicates that the site of damage in presence of oil might also be in the *upstream* process of photosynthesis associated with light absorption. This was confirmed when the lower light harvesting efficiency and light absorption coefficient, which is the fundamental index of cellular light absorption^48,49^, was observed in WAF treated cells compared to the Control.

Light harvesting process of photosynthesis are performed by the pigment-protein assembly called the light harvesting complex^50^. To determine the site of damage in the light absorption processes, we compared the pigment composition and absorption properties of *T. pseudonana* in Control and WAF treatments. Most pigments in WAF treated cells including Chlorophyll *a*, chlorophyll *c*, and diadinoxanthin were similar in concentration compared to the Control, with diatoxanthin and fucoxanthin present in higher levels. The xanthophyll cycle, which in diatoms has been correlated with the thermal dissipation of the excess energy at the antenna side of the photosynthetic apparatus^51^, is found to be sensitive to oxidative stress^52^. In contrary, the de-epoxidation status of the xanthophyll cycle was similar between WAF and Controls, which in turn supports the similar NPQ values observed in these treatments.

Aside from pigments involved in the photoprotective xanthophyll cycle, the major light harvesting pigment composition was not affected under oil exposure, pointing towards proteins. Bopp et al.^22^ and Carvalho et al.^25^ reported that genes and proteins associated with light harvesting proteins were downregulated in *T. pseudonana* when exposed to a mixture of PAHs. To confirm light harvesting proteins to be the site of damage under oil exposure, we conducted additional measurements with the chloroplastic protein synthesis inhibitor, lincomycin. This experiment was conducted under the premise that if proteins associated with light harvesting were damaged by oil exposure, presence of lincomycin would prevent the replacement of damaged proteins by inhibiting the synthesis of new proteins. Furthermore, following the addition of lincomycin, the cultures were moved to lower light levels in order to prevent photo-inhibition and induce rearrangement of light harvesting complex to adapt to the new lower light conditions. This process would be severely affected in case of any protein damage caused by oil exposure. We observed no significant changes to photosynthetic yield but significantly lower functional absorption cross section area in WAF treated *T. pseudonana* compared to the Control. This observation confirmed severe protein damage caused by oil exposure. Moreover, proteome analysis showed a higher abundance of THAPSDRAFT_40669 (Ubiquitin-like domain-containing protein, associated with protein degradation) in WAF relative to Control. This is a clear indication of enhanced protein degradation, presumably a result of the protein damage by oil exposure. Combination of the transfer of cells to a lower light condition and addition of lincomycin led to a significant increase in functional absorption cross section area in the Control treatment, which indicated that the cells rearranged the existing pigments and proteins in order to form a larger light harvesting antennae. Such increase in light harvesting antennae size on transfer to lower light levels has been previously observed^53,54^. A comparatively lower functional absorption cross section area and severe effects on light absorption in WAF + lincomycin compared to the Control + lincomycin indicated inefficient rearrangement of the light harvesting complex and poor absorption and transfer of light energy. Even though the photosynthetic yield was not affected in WAF + lincomycin compared to the Control + lincomycin, which confirmed the *downstream* processes were intact, the amount of energy derived from light absorption transferred *downstream* was severely affected. In addition, proteome analysis revealed several light harvesting proteins such as Lhcr3, Lhcr11 and Lhcf6 that were present in significantly lower abundance in WAF compared to Control, which aligns well with the findings of Bopp et al.^22^ and Carvalho et al.^25^.

Previous studies have shown an ambiguous outcome of oil exposure on the fate of fatty acid synthesis*,* with an increase in lipid synthesis observed in one study^24^, and downregulation of lipid synthesis associated genes observed in the other^25^. We therefore measured the levels of lipid peroxidation caused by oxidative stress in *T. pseudonana* when exposed to WAF and found significant damage to the lipids. Further analysis showed a slight decrease in total lipid content with significant changes in FAME composition upon WAF exposure, with certain components present in similar or lower concentration or entirely absent. This suggest the observed increased lipid peroxidation under oil exposure might preferentially affect certain fatty acids if not all, over a range of time scales. FAME measured in our study could have been derived by membrane (chloroplast and/or mitochondria) damage by depolarization and hydrolysis induced by oxidative stress^52,55^. Lipids account for 31% of the major light harvesting chlorophyll *a*/*b* protein complex^56^, therefore increased lipid peroxidation may have severely destabilized the light harvesting complex by interfering with polypeptide stacking and therefore the light absorption process.

Taken together, all these results suggests that growth inhibition caused by oil exposure could be due to the negative effects observed to the proteins and lipids in the light absorption apparatus and electron transport system of photosynthesis, which in turn reduced the ability of the cell to photosynthesize and therefore produce organic carbon and energy. To test the extent of energy and carbon deprivation caused by oil induced damage to the photosynthetic apparatus, we provided acetate as an external carbon source to *T. pseudonana* in the presence and absence of oil. Acetate served as an alternative carbon source, thereby will alleviate the dependency of the cell on photosynthesis to derive organic carbon and energy. The stimulation of growth in the presence of acetate was relatively lower in WAF compared to Control, however the observed growth was significantly higher than WAF alone and similar to Control only treatment. This response clearly underscores our results that the cells were limited in organic carbon and energy supply due to damage to photosynthetic apparatus caused by oil exposure. Simultaneously, the relatively poor stimulation of growth by acetate in WAF (2.1 fold) compared to Control (3.8 fold) suggests that the ability to catabolize organic carbon, could have been also affected by oil. Pathway enrichment analysis showed that biological function associated with organic substance catabolism were negatively affected in WAF, which backs the observation of poor growth stimulation under acetate supplementation in the presence of oil.

Multiple studies have suggested that exposure to oil induces oxidative stress in the exposed cells, thereby causing severe oxidative damage to the intracellular components^22–25,38^. However, whether the oxidative stress is a primary/direct or secondary/indirect effect of oil exposure remains unknown. By monitoring the oxidative stress in the Control and WAF treatments over a time course, we found that the total ROS levels are very similar between the treatments, however when expressed per cell, the amount of ROS load per cell significantly increases. This effect is primarily due to the inhibition of growth caused by oil exposure, which in turn reduces the cell numbers and thereby increasing the ROS to cell concentration ratio. This along with the observed lipid peroxidation indicates that the oxidative stress is not a primary effect of oil exposure but rather secondary.

Lastly, proteome and pathway enrichment analysis other than supporting the experimental observation also revealed several other processes that were negatively affected. These processes include ferroxidase complex, high-affinity iron permease complex, transmembrane transport activity such as inorganic anion transmembrane transporter activity, ion transmembrane transporter activity, transmembrane transporter complex, cell periphery and plasma membrane, indicating photosynthesis was not the sole target. While the negative effects in several membrane transport related processes aligns with the increased lipid peroxidation observed in the presence of oil, it also suggests that the ability to cells to assimilate and/or exchange ions and molecules both internally amongst the different organelle and externally from the environment could have been compromised. Enrichment analysis performed for cellular component supports this conclusion.

Overall, by using model diatom species *T. pseudonana*, we have developed a process based understanding of how oil affects growth and photosynthesis in phytoplankton. We provide clarity on previous findings^22–25^ that had shown silica uptake is not inhibited and lipid peroxidation is increased under oil exposure. More importantly, we show that the electron transport process and the light harvesting process of photosynthesis were negatively impacted. Through an in depth analysis of light harvesting components and associated processes during oil exposure, we show that the major pigments of the light harvesting pigment-protein complex were not affected, however the respective proteins and membrane lipids were damaged during oil exposure, thus decreasing the amount of light absorbed and therefore the overall energy derived from photosynthetic performance. Our study concludes that the decreased energy production caused by photosynthetic damage, severe detoriation to the several membrane transport processes, and increased spending of available carbon and energy towards protein and lipid repair eventually affected the growth of the diatom. Together with studies conducted by Bopp et al.^22^, Carvalho et al.^23–25^, we provide a detailed mechanism of how oil exposure leads to reduced photosynthesis and growth, and therefore how oil spills can alter phytoplankton community composition.

## Material and methods

*T. pseudonana* (National Center for Marine Algae—CCMP1335) was maintained at 19 °C in f/2 medium at 60 µmol photons m^−2^ s^−1^ and a 12-h/12-h light/dark cycle. Five experiments were conducted under these environmental conditions to understand how oil inhibits *T. pseudonana* growth in a laboratory settings between January and July 2019 in one liter Schott bottles, with no stirring and lids tightly closed to minimize loss of oil by evaporation. Cell density at the start of experiments was of 10^5^ cells mL^−1^. A Water Accommodated Fraction (WAF) of oil was prepared using the CROSERF method^40^. Briefly, 400 µL of Macondo surrogate oil was added per L sterile f/2 growth media and stirred overnight in the dark. Afterwards, it is filtered using 20 µm Teflon sieve to obtain WAF free of large oil droplets. The resultant oil concentration in WAF medium resulted in an average of 2.75 mg/L (± 2.26 mg/L) among all the experiments conducted in this study. Out of the five separate experiments conducted, the first experiment determined the growth and photo-physiology under Control and WAF treatment. In the second experiment, measurements such as proteomics and pathway enrichment analysis, fatty-acid methyl ester analysis, reactive oxygen species content, pigment analysis, silica content, malondialdehyde content and cellular morphology were performed under Control and WAF treatment. In the third experiment, the effects of alkane and PAH component on *T. pseudonana* was determined. The fourth experiment determined the extent of protein damage in the photosynthetic apparatus using chloroplastic protein synthesis inhibitor—Lincomycin. Lastly, the fifth experiment was conducted to determine the extent of carbon and energy deprivation caused by oil exposure by growing *T. pseudonana* in the presence and absence of an external organic carbon source—Acetate in Control and WAF. A complete description of the materials and methods used in this study is provided in the SI Appendix, Materials and Methods.

## Supplementary Information

Supplementary Information 1.

Supplementary Information 2.

Supplementary Information 3.

Supplementary Information 4.

Supplementary Information 5.
