# Supplementary material for: Molecular mechanism of oil induced growth inhibition in diatoms using Thalassiosira pseudonana as the model species
Source: Sci Rep. 2021 Oct 6;11:19831. doi: 10.1038/s41598-021-98744-9 (PMC8494926; doi:10.1038/s41598-021-98744-9)
Supplement: Supplementary file 2 — Supplementary Information 2. [file 41598_2021_98744_MOESM2_ESM.docx]

**Molecular mechanism of oil induced growth inhibition in diatoms using *Thalassiosira pseudonana* as the model diatom species**

Manoj Kamalanathan^a^*, Savannah Mapes^a,^^, Jessica Hillhouse^a^, Noah Claflin^a^, Joshua Leleux^a^, David Hala^a^, Antonietta Quigg^a,b^

^a^Department of Marine Biology, Texas A&M University at Galveston, Galveston, Texas 77553

^b^Department of Oceanography, Texas A&M University, College Station, Texas 77845

^^^*present address*, Virginia Institute of Marine Science, Gloucester Point, VA 23062

*Corresponding author: Manoj Kamalanathan, Department of Marine Biology, Texas A&M University at Galveston, Galveston, Texas 77553

Phone: 409-443-6383

Email: [manojka@tamu.edu](mailto:manojka@tamu.edu)

**Key-words:** Phytoplankton, Diatoms, Oxidative stress, Reactive oxygen species (ROS), Oil, Light harvesting complex, Lipid Peroxidation, Polycyclic Aromatic Hydrocarbons (PAHs)

**Supplementary material 1:** Detailed description of the materials and methods used in this study.

**Materials and methods**

**Experiment no. 1:**

The first experiment was conducted over a period of seven days and consisted of two treatments: Control f/2 medium and WAF, in triplicates. Exponential cultures of *T. pseudonana* were inoculated in 1L of Control and WAF. Growth was monitored by daily cell counts using a Neubauer hemocytometer. Photo-physiological parameters such as maximum quantum yield (*F*_v_/*F*_m_; relative units), relative electron transport rates (*r*ETR_max_; µmol electron m^-2^ s^-1^), and light harvesting efficiency (α; µmol electrons/µmol photons) were measured on dark acclimated (15 mins) cultures using a Pulse-Amplitude–Modulated Fluorescence System (Phyto-PAM; Walz), operated by the software Phyto-Win (v2.13; Walz). A Fluorescence Induction and Relaxation Fluorometer System (Satlantic) was used to measure functional absorption cross-section area (σPSII; Å^2^ quanta^–1^) and QA turnover rates of plastoquinone (τ_PQ_; µs) on dark acclimated (15 mins) cultures. Estimated oil equivalents (EOE) were used to measure the concentrations of oil in WAF treatments daily according to Wade et al. (57). Briefly, the samples were mixed with dichloromethane (DCM) to extract the hydrocarbons. After phase separation, the fluorescence in the DCM layer was measured at 322/376 nm excitation/emission wavelengths using a Shimadzu spectrophotometer (RF-5301PC; Shimadzu, Houston, TX, USA).

**Experiment no. 2:**

This experiment was conducted over 48 hrs and consisted of two treatments, Control and WAF, in triplicates. 48 hr time point was chosen based on our previous study (Kamalanathan et al 2019), which showed that oil concentration reached below detection limits past 4 days. After 48 hrs, cells were harvested for proteomics analysis. In addition, parameters such as protein concentration, respiration rates, fatty acid methyl ester (FAME) analysis, ROS levels, pigment analysis, Malondialdehyde (MDA) content, silica content, cell volume, cell surface area, relative electron transport rates (rETRmax), and non-photochemical quenching (NPQ).

For proteomics, cells were filtered on a glass microfiber filter (Whatman) and proteins were extracted by using MPBio FastProtein Red Matrix kit. The extracted proteins were solubilized with 5% SDS, 50mM Tetraethylammonium tetrahydroborate (TEAB), pH 7.55, final volume 25 µL, followed by centrifugation at 17,000 g for 10 mins. Proteins were reduced by making the solution 20 mM TCEP (Thermo, #77720) and incubated at 65^○^C for 30 mins. Samples were then cooled to room temperature and supplemented with 1 µL of 0.5 M iodoacetamide acid and incubated in dark for 20 mins. 12% phosphoric acid (2.75 µL) was added to the protein solution. Binding buffer was prepared by adding 100 mM TEAB to 90% methanol and the pH was adjusted to 7.1. 165 µL of binding buffer was then added to the protein solution and transferred to S-Trap spin column (protifi.com) and centrifuged for 30 secs at 4,000 g. The spin column was washed with 400 µL of binding buffer and centrifuged two more times. Trypsin was then added to the protein mixture in a ratio of 1:25 in 50 mM TEAB (pH=8), and incubated at 37^○^C for 4 hrs. Peptides were then eluted with 50mM TEAB (80 µL), followed by 0.2% formic acid (80 µL), and finally with 50% acetonitrile and 0.2% formic acid (80 µL). The combined peptide solution was then dried in a speed vacuum and re-suspended in 2% acetonitrile, 0.1% formic acid, 97.9% water and stored in an autosampler vial.

NanoLC MS/MS Analysis. A nanoflow liquid chromatography-tandem mass spectrometry (nanoLC-MS/MS) and a nano-LC chromatography system (UltiMate 3000 RSLCnano, Dionex), coupled on-line to a Thermo Orbitrap Fusion mass spectrometer (Thermo Fisher Scientific, San Jose, CA) through a nanospray ion source (Thermo Scientific) was used to analyze the peptide mixtures. A C18 PepMap100 (300um X 5mm, 5um particle size, Thermo scientific) was used as trap column, and an Acclaim PepMap 100 (75um X 25 cm, Thermo scientific) was used as an analytical columns. Samples (1 µL in solvent A) were injected onto the trap column and subsequently eluted (400 nL/min) by gradient elution onto the C18 column as follows: isocratic at 2% B, 0-5 min; 2% to 45% B, 2-37 min; 45% to 90% B, 37-40 min; isocratic at 90% B, 40-45 min; 90% to 2%, 45-47 min; and isocratic at 2% B, 47-60 min.

XCalibur, version 2.1.0 (Thermo Fisher Scientific) was used to acquire all LC-MS/MS data in positive ion mode using a top speed data-dependent acquisition (DDA) method with a 3 sec cycle time. Survey scans (m/z 370-1570) were acquired in at 120,000 resolution (at m/z = 400) in profile mode, with maximum injection time of 56 msec, AGC target of 600,000 ions and S-lens RF level set to 60. After isolation in the quadrupole with a 1.6 Da isolation window, HCD MS/MS acquisition was performed in centroid mode using rapid scan rate with detection in the ion trap, using the following settings: collision energy = 32%; AGC target 200,000 ions; maximum injection time 56 msec. Monoisotopic precursor selection (MIPS) and charge state filtering with charge states 2-6 were kept on. Precursor ions were removed using dynamic exclusion, with a +/- 10 ppm mass tolerance, for 15 sec after acquisition of one MS/MS spectrum.

Database Searching- Proteome Discoverer (Thermo Fisher, version 1.4.1.14) was used for tandem mass spectra were extraction and charge state deconvolution. Deisotoping was not performed. All spectra were searched against a Thalassiosira database concatenated to the cRAP database (version 02-26-2019) using Sequest. Search parameters include a parent ion tolerance of 5 ppm and a fragment ion tolerance of 0.60 Da with Trypsin specified as the enzyme, allowing for two missed cleavages and fixed modification of carbamidomethyl (C) and variable modifications of oxidation (M). The peptide levels were then normalized to total spectra. Student’s t-test was performed to determine the peptides that were present in significantly differential abundance. Statistically significant differentially abundant peptides were selected and ranked (adjusted p-value < 5%, FDR) and used for pathway enrichment analysis.

Pathway enrichment analysis- Gene ID’s for each peptide were determined manually for each of the statistically significant differentially abundant peptides. Peptides were separated into negatively and positively affected depending on fold change values and ranked based on decreasing order of significance. Only one peptide was upregulated in WAF, hence only negatively affected peptides were used for pathway enrichment analysis. The analysis was performed as per methods described by Reimand et al. (58). Briefly, pathway enrichment was performed using g:Profiler (https://biit.cs.ut.ee/gprofiler/gost) with *Thalassiosira pseudonana* CCMP 1335 (same as the one used in our experiment) was chosen as the organism, “All known genes” as the statistical domain scope, and significance threshold set at Benjamini-Hochberg FDR. Custom GMT file was used as the data source for the pathway enrichment analysis (Supplementary material 2). The enrichment map was then visualized via Cytoscape (v 3.8.0), with an FDR q-value cut-off of 0.05 and the default connectivity parameters. Overall, only one gene set (KEGG:00195) was missing from the customized GMT file while building the enrichment map using Cytoscape. However, when the default cellular component (CC) gene ontology (GO) source gmt file available in g:Profiler was used for building the enrichment map, 85 gene sets were missing. This could be primarily due to lack of GO information available with regards to CC for *Thalassiosira pseudonana* CCMP 1335.

Protein concentration was measured using a Micro BCA protein assay kit. Briefly, 50 mL of samples were centrifuged, the pellet was treated with 90% methanol for cell lysis. The methanol extract was then used to measure protein content using the kit. Respiration rates were measured using a Clark-type oxygen electrode (Hansatech, Norfolk, United Kingdom), as per methods described in Kamalanathan et al. (59).

Lipid extraction was carried out using a modified version of the procedure from Folch et al. (60). Samples (200 mL) were filtered on a glass microfiber filter (Whatman). The filtered sample was briefly vortexed in a solution of 2 mL chloroform, 1mL methanol, and 0.5 mL milliQ-water and sonicated for 10 mins then flushed with N_2_ gas for 30 sec. After phase separation followed by centrifugation at 125 g for 3 minutes, chloroform extract was collected and evaporated under a steady flow of N_2_ gas until only lipid residue remained. The lipid was transesterified by adding an acid catalyst; 1.5 mL of (1.3 M) Boron trifluoride-methanol (BF_3_/CH_3_OH) with 0.5 mL hexanes to extract the FAME. After addition of N2 gas, vials were capped and vortexed for 30 sec before being placed in the oven at 85^o^C for 90 mins with intermittent mixing. This was followed by addition of 2 mL hexanes, 0.5 mL milliQ-water, and 10 μL (d33-Heptadecanoic acid) internal standard. The hexane extract was filtered and evaporated in a SavantTM SPD121P SpeedVacTM Concentrator (Thermo Scientific). The resulting residue was reconstituted with 0.5 mL milliQ-water, and 0.5 mL DCM. After removal of water from the sample with sodium sulphate, the FAME were reconstituted for a final time with 1mL DCM and stored in freezer at -20^o^C until gas chromatography and mass spectrometry (GC/MS) analysis using a Hewlett Packard HP-6890 gas chromatograph coupled to an Agilent 5973 mass spectrometer. Samples were injected in splitless mode (1µL) equipped with a CP-Sil 88 (J&W Scientific) capillary column (100 m x 0.25 mm i.d.; 0.20 µm film thickness). Helium was the carrier gas at a flow rate of 1.0 mL/min. Temperatures at the front inlet and the MS interface were set at 250 and 260°C, respectively. Following injection of the sample, the GC oven was programmed at 100°C and held for 5 min, then ramped up to 180°C at 8°C/min, held for 9 min, and finally ramped up to 230°C at 1°C/min and then held for 15 mins. FAME were quantified using the deuterated standards and adjusted to percent recovery of the standards.

For ROS measurements, samples were taken every hour for the first six hours and then once at 24 and 48 hours. ROS levels were quantified using modifications of methods described by Rijstenbil, (16). Briefly, 1 mL of samples were stained with 10 mM of 2',7'-dichlorofluorescien diacetate (DCFH-DA) for 60 mins in complete darkness. The samples were then centrifuged at 10,000 g for 5 mins, following which the supernatant was discarded, and the stained cells were re-suspended with fresh f/2 media. The fluorescence was then measured at 488/520 nm excitation/emission using BioTek CytationTM 5 imaging reader controlled by Gen52.09 software (USA). The results were then expressed as relative fluorescence per ml and per cell.

Pigment analysis was performed two ways. First, the absorption coefficient was measured using UV-Visible 2501 Shidmazu spectrophotometer fitted with an integrating sphere according (61, 62). Briefly, 200 mL of samples were filtered onto polycarbonate filters (0.25 µm) and the absorbance was measured from 400 to 750 nm. A seawater blank was used to substrate background absorbance. Quantification of pigments was performed using reverse-phase HPLC analysis according to procedures in Zhao and Quigg (21). The instrument includes a binary gradient pump (Shimadzu dual LC10-ATvp and Controller SCL-10Avp), temperature controlled autosampler (Shimadzu SIL 10-Avp) with a 500 mL injection loop, column oven (Shimadzu CTO-10AS vp), and photodiode array detector (PDA, Shimadzu SPD-M10A vp; 200 to 800 nm). Experimental samples were filtered onto GF/F glass fiber filters (Whatman) and frozen at -80°C until extraction in cold 100% acetone with 100 µL synthetic carotenoid b-apo-89-carotenal (internal standard) overnight. Before injection to the HPLC, samples were pre-filtered through a 0.2 mm PTFE (Gelman Acrodisc) filter. 300–400 mL extracted samples mixed with 1.0 mol L21 ammonium acetate (ion-pairing solution) in a ratio of 4 (extracted sample):1(ammonium acetate) were added to the vials then placed in the autosampler rack for HPLC analysis. Pigment peaks were identified based on retention time and pigment spectra shape obtained from liquid standards (DHI, Hørsholm, Denmark). Chlorophyll *a* (chl *a*), chlorophyll *c* (chl c1c2), β-carotene, fucoxanthin, diadinoxanthin and diatoxanthin were detected by HPLC analysis.

Malondialdehyde (MDA) content, an index of cumulative ROS toxicity, was determined as described in Li et al. (14). Briefly, 50 mL of culture was filtered and homogenized with 0.8 mL of 20% (w/v) trichloroacetic acid (TCA). The solution was then centrifuged at 13,000 g (10 min), and 0.35 mL of the supernatant was collected and mixed with 0.35 mL of thiobarbituric acid reagent (0.5% in 20% TCA) and incubated at 90°C for 30 min and then cooled on ice. The absorbance was then measured at 532 and 600 nm and MDA content calculated with extinction coefficient of 6.45 µM cm^−1^.

Silica content was determined using methods described by Smith-Harding (63). Briefly, 40 mL culture filtered onto pre-rinsed 0.2 µM polycarbonate filters were incubated with 18 ml of 0.5% sodium carbonate (Na_2_CO_3_) at 85°C for 2 hours. The samples were then cooled and neutralized with 0.5 N HCl (3.25 mL) and the volume was adjusted to 25 mL with distilled water (64). The silica content was then determined according to Strickland and Parsons (65) using Na_2_SiO_3_ solution as standard.

Cell volume and surface area were microscopically estimated using procedures described in Kamalanathan et al. (66) using the following formulae:

Volume of the cell = πr^2 h

Surface area of the cell =2πrh +2πr^2

where r is the radius and h is the height of the cells.

*r*ETR_max_ and NPQ were determined using methods described in Kamalanathan et al. (66) using a Pulse-Amplitude–Modulated Fluorescence System on dark adapted (15 mins) cells as described above.

**Experiment no. 3:**

A short exposure experiment (1 hr) in which *T. pseudonana* was exposed to an alkane mix (C8-20) at a concentration of 100 ng. mL-1 and a mixture of PAHs at a concentration of 10 ng.mL-1 for 60 mins. Two other treatments, Control with no additions, and Control+DCM with equal volume of DCM served as controls for this experiment. After incubation, cells were dark adapted for 15 mins before *r*ETR_max_ was measured using a Phyto-PAM (see above). The concentrations of alkane and PAHs mix used were 6-7 fold lower than the average concentrations in the WAF on the initial day of the experiment conducted by Kamalanathan et al. (27).

**Experiment no. 4:**

This experiment was conducted over a 24 hrs with four treatments (Control, Control+Lincomycin, WAF, WAF+Lincomycin) in triplicates. After 100 mins of incubation in Control f/2 medium and WAF, the cultures were split in two halves and 2 mM final concentration of lincomycin was added to one of them, which are termed as Control+Lincomycin and WAF+Lincomycin treatments respectively. The Lincomycin concentration used was chosen based on the findings of Heraud et al., (67). Immediately after addition of lincomycin all the treatments were transferred to a lower light level (10 µmol photons m^-2^ s^-1^). Relative functional absorption cross-section area (σPSII’; Å^2^ quanta^–1^) and yield (*F*_v_’/*F*_m_’), were then measured at 0, 20, 40, 60, 80, 120, 180, 240 and 300 mins using a Fluorescence Induction and Relaxation Fluorometer System (Satlantic). Light absorption was approximated by dividing σ’ with *F*_v_’/*F*_m_’ for each time points according to Xu et al., (68).

**Experiment no. 5:**

The last experiment was conducted over a 48 hrs with four treatments (Control, Control+Acetate, WAF, WAF+Acetate). 0.5 g.L-1 sodium acetate was added to both Control+Acetate and WAF+Acetate (69). Growth was monitored in all the treatments through cell counts using a Neubauer hemocytometer.

**Statistics**

Student’s t-test and one-way ANOVA with Tukey’s test were performed using software package GraphPad Prism (v7.04; https://www.graphpad.com/scientific-software/prism/).
