# Supplementary material for: Molecular mechanism of oil induced growth inhibition in diatoms using Thalassiosira pseudonana as the model species
Source: Sci Rep. 2021 Oct 6;11:19831. doi: 10.1038/s41598-021-98744-9 (PMC8494926; doi:10.1038/s41598-021-98744-9)
Supplement: Supplementary file 4 — Supplementary Information 4. [file 41598_2021_98744_MOESM4_ESM.docx]

**Supplementary Table 1:**

List of statistically significant differentially abundant peptides, with gene and protein ID, fold enrichment, p-values and FDR adjusted p-value used for pathway enrichment analysis.

| **Protein ID** | **Gene id** | **p-values (t-test)** | **FDR adjusted p values** | **Fold enrichment** |
| --- | --- | --- | --- | --- |
| THAPSDRAFT_268009 | 7442890 | 0.002656 | 0.039761 | 0.25267 |
| PPC1 | 7452058 | 0.002795 | 0.039761 | 0 |
| THAPSDRAFT_25918 | 7446832 | 0.004732 | 0.039761 | 0 |
| psaD | 4524841 | 0.004732 | 0.039761 | 0 |
| THAPSDRAFT_35934 | 7445547 | 0.007099 | 0.039761 | 0 |
| THAPSDRAFT_24512 | 7445847 | 0.007543 | 0.039761 | 0 |
| THAPSDRAFT_bd718 | 7447330 | 0.008984 | 0.039761 | 0 |
| glsF | 7453132 | 0.009926 | 0.039761 | 0 |
| psbB | 4524802 | 0.010625 | 0.039761 | 0.325676 |
| THAPSDRAFT_262506 | 7450043 | 0.011202 | 0.039761 | 0 |
| atpA | 7452400 | 0.011955 | 0.039761 | 0.069658 |
| THAPSDRAFT_270396 | 7451396 | 0.01211 | 0.039761 | 0.576077 |
| THAPSDRAFT_26146 | 7441736 | 0.013579 | 0.039761 | 0 |
| gapC3 | 7451762 | 0.014271 | 0.039761 | 0.514326 |
| THAPSDRAFT_24932 | 7451589 | 0.015378 | 0.039761 | 0 |
| THAPSDRAFT_26224 | 7451415 | 0.016484 | 0.039761 | 0 |
| THAPS_14106 | 7444148 | 0.016484 | 0.039761 | 0 |
| THAPS_25409 | 7444818 | 0.016484 | 0.039761 | 0 |
| TPI2 | 7444271 | 0.016484 | 0.039761 | 0 |
| THAPSDRAFT_264361 | 7449984 | 0.016484 | 0.039761 | 0 |
| Lhcr3 |  | 0.016484 | 0.039761 | 0 |
| PbgD | 7452450 | 0.016484 | 0.039761 | 0 |
| THAPSDRAFT_10383 | 7449986 | 0.016484 | 0.039761 | 0 |
| THAPSDRAFT_20812 | 7445018 | 0.016484 | 0.039761 | 0 |
| THAPSDRAFT_22108 | 7442070 | 0.016484 | 0.039761 | 0 |
| Lhcr11 | 7450213 | 0.016484 | 0.039761 | 0 |
| THAPSDRAFT_267922 | 7451528 | 0.016484 | 0.039761 | 0 |
| GMD1 | 7448453 | 0.016484 | 0.039761 | 0 |
| RS5 | 7451203 | 0.016484 | 0.039761 | 0 |
| THAPSDRAFT_260835 | 7444164 | 0.01704 | 0.039761 | 0 |
| THAPSDRAFT_40669 | 7451773 | 0.020702 | 0.044049 | 3.046086 |
| Lhcf6 | 7444045 | 0.020988 | 0.044049 | 0 |
| THAPSDRAFT_270229 | 7452334 | 0.020988 | 0.044049 | 0 |
| THAPSDRAFT_11697 | 7449481 | 0.021651 | 0.044049 | 0 |
| THAPSDRAFT_bd1611 | 7447383 | 0.022455 | 0.044049 | 0.107051 |
| rbcS | 4524792 | 0.022654 | 0.044049 | 0 |
| AAT2 | 7451457 | 0.023316 | 0.044111 | 0 |
| AMS1 | 7441903 | 0.024015 | 0.044238 | 0.222708 |
| THAPSDRAFT_bd1154 | 7447314 | 0.025987 | 0.045477 | 0 |
| THAPSDRAFT_1786 | 7449060 | 0.025987 | 0.045477 | 0 |
| THAPSDRAFT_20603 | 7451524 | 0.027968 | 0.046614 | 0 |
| THAPSDRAFT_3880 |  | 0.027968 | 0.046614 | 0 |
| GLNN | 7448923 | 0.030024 | 0.047939 | 0.09358 |
| THAPSDRAFT_bd1863 | 7449608 | 0.030133 | 0.047939 | 0 |
